# Effects of *THBS3*, *SPARC *and *SPP1 *expression on biological behavior and survival in patients with osteosarcoma

**DOI:** 10.1186/1471-2407-6-237

**Published:** 2006-10-05

**Authors:** Cristiane A Dalla-Torre, Maisa Yoshimoto, Chung-Hae Lee, Anthony M Joshua, Silvia RC de Toledo, Antônio S Petrilli, Joyce AD Andrade, Susan Chilton-MacNeill, Maria Zielenska, Jeremy A Squire

**Affiliations:** 1 Department of Pediatrics, Instituto de Oncologia Pediátrica (IOP-GRAACC), Universidade Federal de São Paulo, Escola Paulista de Medicina, São Paulo 04023-062, Brazil; 2 Department of Morphology, Division of Genetics, Universidade Federal de São Paulo, Escola Paulista de Medicina, São Paulo 04023-900, Brazil; 3 Applied Molecular Oncology, Ontario Cancer Institute, Princess Margaret Hospital, Toronto, Ontario M5G 2M9, Canada; 4 Department of Pediatric Laboratory Medicine, The Hospital for Sick Children, Toronto, Ontario M5G 1X8, Canada; 5 Department of Laboratory Medicine and Pathobiology, University of Toronto, Toronto, Ontario M5G 2M9, Canada

## Abstract

**Background:**

Osteosarcoma is a very aggressive tumor with a propensity to metastasize and invade surrounding tissue. Identification of the molecular determinants of invasion and metastatic potential may guide the development of a rational strategy for devising specific therapies that target the pathways leading to osteosarcoma.

**Methods:**

In this study, we used pathway-focused low density expression cDNA arrays to screen for candidate genes related to tumor progression. Expression patterns of the selected genes were validated by real time PCR on osteosarcoma patient tumor samples and correlated with clinical and pathological data.

**Results:**

*THBS3*, *SPARC *and *SPP1 *were identified as genes differentially expressed in osteosarcoma. In particular, *THBS3 *was expressed at significantly high levels (p = 0.0001) in biopsies from patients with metastasis at diagnosis, which is a predictor of worse overall survival, event-free survival and relapse free survival at diagnosis. After chemotherapy, patients with tumors over-expressing *THBS3 *have worse relapse free survival. High *SPARC *expression was found in 51/55 (96.3%) osteosarcoma samples derived from 43 patients, and correlated with the worst event-free survival (p = 0.03) and relapse free survival (p = 0.07). Overexpression of *SPP1 *was found in 47 of 53 (89%) osteosarcomas correlating with better overall survival, event-free survival and relapse free survival at diagnosis.

**Conclusion:**

In this study three genes were identified with pattern of differential gene expression associated with a phenotypic role in metastasis and invasion. Interestingly all encode for proteins involved in extracellular remodeling suggesting potential roles in osteosarcoma progression. This is the first report on the *THBS3 *gene working as a stimulator of tumor progression. Higher levels of *THBS3 *maintain the capacity of angiogenesis. High levels of *SPARC *are not required for tumor progression but are necessary for tumor growth and maintenance. *SPP1 *is not necessary for tumor progression in osteosarcoma and may be associated with inflammatory response and bone remodeling, functioning as a good biomarker.

## Background

Osteosarcoma (OS) is a rare tumor of children and young adults. The peak of incidence occurs in the second decade of life [1]. In Brazil, it is estimated that a total of 350 new cases of OS are diagnosed per year [2], with metastasis in 20.9% of the cases. The presence of metastases at diagnosis is the most significant isolated factor influencing survival in this group [3]. Lung metastases appear in almost 40 to 50% of the patients with OS, and are the major cause of death [4]. The degree of necrosis following definitive surgery is a consistent prognostic factor [5].

Metastases consist of a series of sequential steps, all of which must be successfully completed. These include shedding of cells from a primary tumor into the circulation, survival of the cells in the circulation, arrest in a new organ, extravasations into the surrounding tissue, initiation and maintenance of growth, and vascularization of the metastatic tumor [6]. The successful metastatic cells must possess a set of particular characteristics that are different from non-metastatic tumor cells, enabling them to complete each step in the metastatic sequence [7].

Knowledge on the biology of the metastatic process is limited. Some molecular factors have been identified as contributing to the formation of detectable metastases, and additional factors hopefully will be soon identified using microarray expression profiling [6]. The pathway-focused GEArray^® ^is a useful way to perform both highly sensitive and precise large-scale analysis of the expression level of various genes [8,9]. The advantage of using this technology is that a defined set of genes closely-related to a specific process, biological behavior or biomarker can be screened with limited sample material, as observed by [10-12]. Through these comparisons, a list of genes differentially expressed and potentially important for the process is easily derived that can be validated by other techniques. The biomarkers may become tools for establishing early diagnosis and designing therapeutic targets in specific cancers.

By comparing the highly metastatic K7M2 and K12 murine osteosarcoma cell lines using cDNA microarray technology, Khanna *et al*. [13] identified 10 potential genes involved in the metastatic process in osteosarcoma: *ITGB4 *(integrin β4), *CTGF *(connective tissue growth factor), *ITGA5 *(integrin α5), *VIL2 *(ezrin), *ITGB2 *(integrin β2), *LGALS3 *(galectin-3), *ADAM8 *(a disintegrin and metalloprotease domain 8), *CLU *(clusterin), *FARP1 *(*FERM*, *RhoGEF*, and pleckstrin domain protein 1 isoform 1) and *CP *(ceruloplasmin). These genes are involved in motility/cytoskeleton, heterotypic adherence and/or angiogenesis [13].

Genes identified in this work include those not described previously in OS as well as potentially novel metastasis-associated genes. Functional studies suggest that 10 genes associated with motility/cytoskeleton, heterotypic adherence, and angiogenesis are most likely associated with differences in the metastatic behavior of the high and low metastatic OS models.

In this study, we used the pathway-focused GEArray^® ^technique to screen genes related to tumor progression. *THBS3*, *SPARC *and *SPP1 *were identified by cDNA array as genes differentially expressed in osteosarcoma. In addition, expression of selected genes was significantly correlated with clinico-pathological data.

## Methods

### Sample collection

Seventy-six OS tumor samples were obtained at the Instituto de Oncologia Pediátrica, Grupo de Apoio ao Adolescente e à Criança com Câncer/Universidade Federal de São Paulo (IOP/GRAACC/UNIFESP, São Paulo, SP, Brazil), with informed patient consent and approval of the institutional research ethics board (0261/03). Tissue sections were stained and subjected to standard histopathological evaluation to determine tumor content, as well as the proportion of necrosis and the pathological grade. Tumor specimens were stored at -80°C until a tumor-rich tissue had been selected for RNA extraction, according to the histological assessment by the pathologist. Clinical data for each case was obtained from the medical records.

### Diagnostic staging and treatment

All patients younger than 25 years of age with high-grade OS of the extremities (metastatic and non-metastatic) were prospectively enrolled onto the Brazilian Osteosarcoma Treatment Group study IV [3]. In study IV, patients received carboplatin (500 mg/m^2 ^intravenous infusion on day 1 of weeks 0, 3, 6, 17, and 26) and cisplatin (100 mg/m^2 ^intravenous infusion on day 1 of weeks 0, 3, 6, 11, and 20). Doxorubicin was administered either at a dose of 30 mg/m^2 ^in short-term intravenous infusion on days 1 and 2 of weeks 0, 3, 6, 14, 17, and 23 in the initial phase of the study or at a dose of 35 mg/m^2^, following the same schedule administered in a previous group with dexrazoxane. Intravenous infusion infusions of ifosfamide at 3 g/m^2 ^and mesna, as described [3] were added on days 1, 2, and 3 and on weeks 11, 14, 20, and 26, respectively. The orthopedics team in collaboration with the pediatric oncology team determined the appropriate surgical procedure for each patient. Non-conventional endoprosthesis, resection of expendable bones, plaques, and bone graft fixation (autograft or bone bank) were used. Whenever possible, all pulmonary metastases were surgically removed, after resection of the primary tumor.

### RNA preparation

Total RNA from OS tumors was isolated using the TRIzol^® ^Reagent (Invitrogen) and re-suspended in sterile DEPC water. The amount of RNA was determined by spectrophotometry and sample integrity was monitored through visualization of ribosomal RNAs (28S and 18S) by electrophoresis.

### Gene expression profiling

Pathway-focused expression profiling was performed using a subset of 15 tumors: 9 samples from patients with metastatic disease at diagnosis (3 initial biopsy specimens, 3 primary bone tumor resection specimens, and 3 biopsies of metastasis detected at diagnosis); and 6 samples from patients classed as having non-metastatic disease at diagnosis (3 initial biopsy specimens and 3 primary bone tumor resection specimens). For each group, a pool of an equal amount of RNA from 3 tumor samples from each phase of disease was used. In addition, a pool of RNA derived from osteoblastic cellular preparations from two bone resections unrelated to a pathological condition served as a tissue-specific expression control.

GEArray^® ^Q Series pathway arrays containing 112 sites each (3 blanks, 3 negative reference spots, 10 endogenous genes, and 96 pathway-specific human genes) were obtained from SuperArray Bioscience Corporation (Frederick, USA). The GEArray^® ^Q Series Human Tumor Metastasis Gene Array, Chemokines and Receptors Gene Array and Angiogenesis Gene Array were designed to assess the different expression levels of 96 genes that either interact with or are controlled by each specific biological pathway. Protocol and data analysis of arrays were performed according to the manufacturer's instructions and the gene list and array position for expression profiling are available at Superarray Bioscience Corporation website [14].

Biotin-labeled cDNA were generated with total RNA (1μg) from corresponding samples using GEArray^® ^Ampolabelling LPR Kit (SuperArray Bioscience Corporation^®^). The cDNA probes were then denatured, and hybridized in GEAhyb solution to nylon membranes spotted with gene-specific fragments. Hybridization was performed over-night at 60°C with continuous agitation. Membranes were washed twice in 2 × SSC/1%SDS for 15 minutes each, followed by another wash in 0.1 × SSC/0.5%SDS for 15 minutes. Hybridized probes were visualized with the CPD-Star Chemiluminescent Detection Kit (SuperArray Bioscience Corporation^®^).

Array images were recorded using Kodak X-OMAT LS film (Amersham Biosciences, Piscataway, NJ, USA) and SprintScan 35 Plus (Polaroid Canada Inc., Mississauga, ON, Canada). Images were converted into grayscale and 16-bit tiff format, and the numerical data representing the expression levels of each gene on the arrays were quantified using the GEArray^® ^Expression Analysis Suite (SuperArray Bioscience Corporation^®^). Raw data were intra-normalized with the mean signal intensity of the *ACTB *gene after subtracting the mean intensities of the negative control spots (pUC18 plasmid DNA and blank spots. Inter-normalization was performed with control normal bone signal intensities.

The change in the level of a given gene transcript from one membrane/experiment was estimated by comparing the signal intensities of paired specimens, such as: (1) biopsy sample from patients with metastasis at diagnosis *versus *normal bone; (2) primary tumor resection sample from patients with metastasis at diagnosis *versus *normal bone; (3) metastatic sample from patient with metastasis at diagnosis *versus *normal bone; (4) biopsy sample from patients with non metastasis at diagnosis *versus *normal bone; (5) primary tumor resection sample from patients with non metastasis at diagnosis *versus *normal bone. The differentially expressed genes were determined based on two criteria: gene performance outline filtering was applied to exclude the lower intensity or absent values, and only genes with fold changes larger than -2 and +2 were considered for increased/decreased expression.

The expression datasets presented in this publication have been deposited in NCBIs Gene Expression Omnibus [15] and are accessible through GEO Series accession number GSE5045.

### Quantitative real-time PCR

From 43 different patients, 20 biopsies, 22 primary tumors, and 19 metastases specimens were included for real-time PCR analysis. Due to low quantity of RNA, the relative quantification of *THBS3 *was performed on 52 samples derived from 40 patients, *SPARC *was performed on 55 samples derived from 43 patients, and *SPP1 *was performed on 53 samples derived from 41 patients.

Total RNA was reverse-transcribed using the Gene Amp^® ^Gold RNA PCR Core Kit (Applied Biosystem^®^) according to the manufacturer's instructions. The RT^2 ^PCR Primer Sets (SuperArray Bioscience Corporation^®^) for human *THBS3*,*SPARC*, *SPP1 *(target genes) and β-actin (*ACTB *– endogenous gene) were used. Real-time PCR amplification and data analysis were performed using the ABI Prism 7900 and 7700 HT Sequence Detector System (Applied Biosystems^®^). Each sample was mixed with 24μl of master mix (SYBR^® ^Green PCR Master Mix – Applied Biosystems^®^). The PCR conditions were 2 minutes at 50°C, 10 minutes at 95°C, followed by 40 cycles of 15 seconds at 95°C and 1 minute at 60°C. Experiments were performed in triplicate, for both the target and the endogenous genes. A no-template control was included in each amplification reaction. The PCR efficiencies of the four genes were comparable (≥ 95%).

### Real-time PCR data analysis

To determine the relative quantification of gene expression, the comparative Ct (cycle threshold) method was used [16]. Briefly, this method uses arithmetic formulas to determine relative quantification. To normalize the varying cDNA quantities, an endogenous control gene (β-actin – *ACTB*) was run concurrently with the target gene. For each sample, the Ct was determined for both the target gene and the endogenous gene (median of the 3 reactions). The ΔCt was determined by subtracting the *ACTB *Ct from the specific target gene Ct. Identical calculations were made for a known positive control reference sample (QPCR Human Reference Total RNA, Stratagene, La Jolla, CA, USA). The ΔCt of the control reference was then subtracted from the ΔCt of the target gene, yielding the ΔΔCt, and the relative quantitative value was expressed as 2^-ΔΔCt^.

### Statistical analysis

Data analysis was performed using Minitab Statistical Software (14.0). Clinical variables correlated with gene expression were: age at diagnosis, gender, histological OS subtype, site of the primary disease, metastatic status at diagnosis, response to chemotherapy, and type of samples (initial biopsy, primary bone tumor resection and metastasis sample present at diagnosis). Overall survival was defined as the time from diagnosis until the date of either the last follow-up or death. The duration of event-free survival was defined as the time from diagnosis until the date of relapse or death. If these patients had metastatic disease at diagnosis, the event was considered as time 0. For the relapse free survival analysis, the duration was defined as the time from diagnosis until the occurrence of metastasis or local relapse.

All samples from each biopsy, primary tumor resection, and at metastasis were evaluated using gene expression profile findings, and these variables were correlated with survival. Overall survival, event-free survival and relapse-free survival curves were generated by applying the Kaplan-Meier method, and were then compared by the log-rank test. Continuous data were evaluated and compared using ANOVA, Mann-Whitney or Mood's Median test. Categorical data were studied using *Chi*-square or Fisher exact tests. Statistical significance was taken as p < 0.05.

## Results

A summary of the clinico-pathological parameters for each case is detailed in Table 1. There was no significant difference in relative expression levels of *THBS3*, *SPARC *and *SPP1 *when the initial biopsy specimens, the primary bone tumor resection specimens, and the biopsies of metastasis were compared. Therefore we examined differential levels of gene expression of all three genes in primary tumor RNA (from both biopsy specimens and bone tumor resections) to assess their utility as biomarkers of prognosis. Thus in these analyses expression levels in primary tumor RNA were used as predictors of overall survival, event-free survival and relapse-free survival.

### Pathway-focused GEarray^® ^analysis

Samples from biopsy, primary tumor resection and metastatic site (metastatic group only) from the metastatic and non-metastatic groups were each processed for gene expression analysis using the GEArray^® ^Q Series Human Tumor Metastasis Gene Array, Chemokines and Receptors Gene Array, and Angiogenesis Gene Array. Thirty-two genes from the angiogenesis membrane, 51 from the tumor metastasis membrane and 25 genes from the chemokine membrane were identified as differentially expressed. The values of the selected *THBS3*, *SPARC*, *and SPP1 *genes are presented in Table 2. Correlation coefficients did not detect any relationship between the relative expression levels of these genes considered at any time point or in combination (-0.11 < Adjusted R^2 ^< 0.14).

### THBS3 mRNA expression in osteosarcoma patients

**Figure 1:**
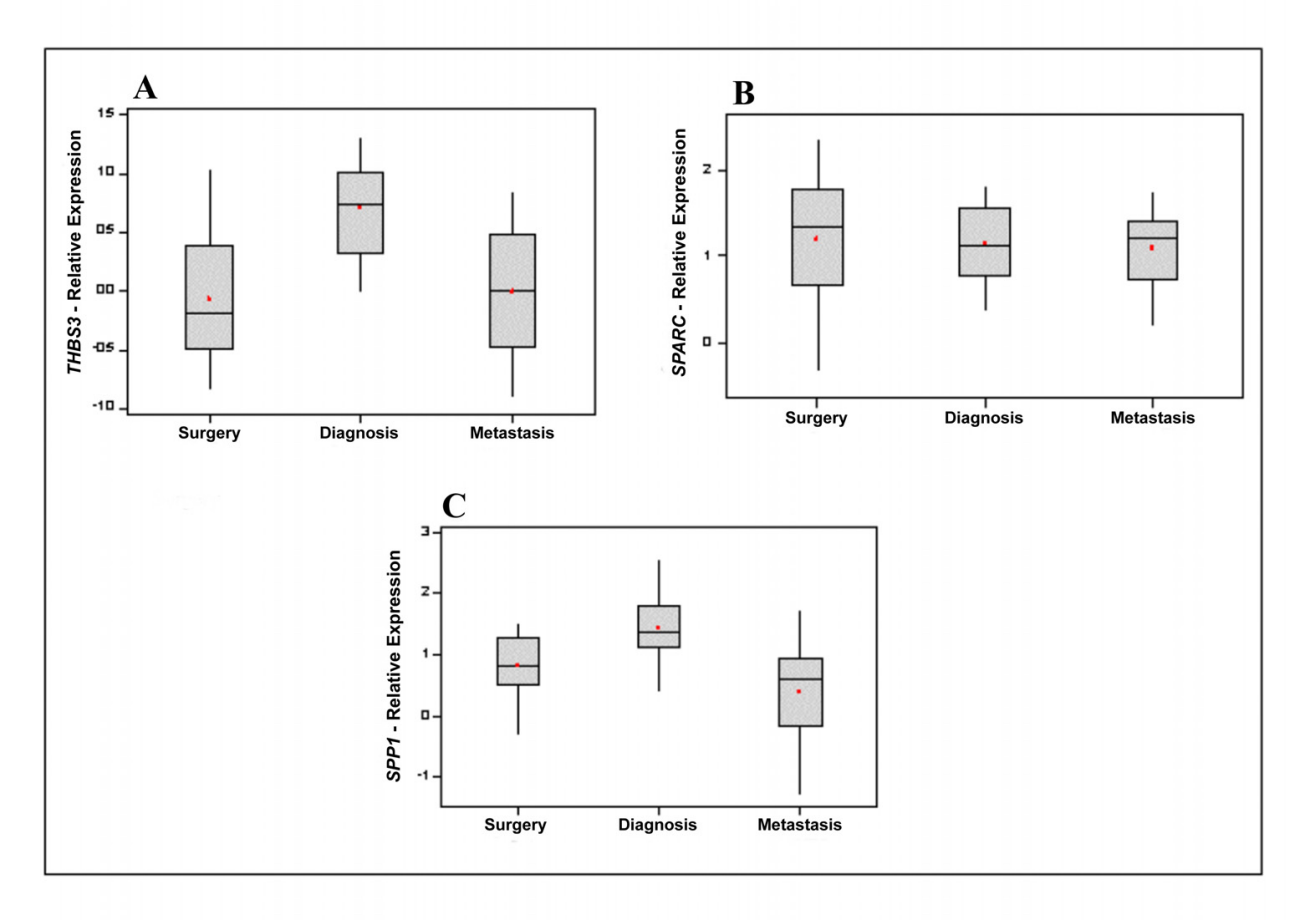
Relationship between gene expression and clinical evolution of disease. The box plot shows the median, upper and lower quartiles and minimum and maximum values for related gene expression. **A- **Relative expression of *THBS3 *in groups of OS patients represented by samples obtained from the biopsy **diagnosis **(n = 16), primary tumor **surgery **(n = 19) and **metastases **(n = 17) ; **B- **Relative expression of *SPARC *in groups of OS patients represented by samples obtained from the biopsy **diagnosis **(n = 26), primary tumor **surgery **(n = 16) and **metastases **(n = 13); **C- **Relative expression of *SPP1 *in groups of OS patients represented by samples obtained from the biopsy **diagnosis **(n = 16), primary tumor **surgery **(n = 20) and **metastases **(n = 17).

To confirm *THBS3 *over-expression, we performed quantitative real-time PCR on 52 samples from 40 patients. Of the 52 tumors analyzed, 30 (58%) showed high expression of *THBS3 *and 22 samples (42%) showed low expression of *THBS3*. A comparison of the *THBS3 *expression levels in biopsy *versus *primary tumor resection or metastatic samples showed significant differences (p = 0.0001, p = 0.0001, respectively). However, this correlation was not significant in primary tumor resection samples *versus *metastases (p = 0.72) (Figure 1A). In addition, different levels of *THBS3 *expression were observed when biopsy and surgical samples from the same patients were compared (n = 7) (p = 0.031).

**Figure 2:**
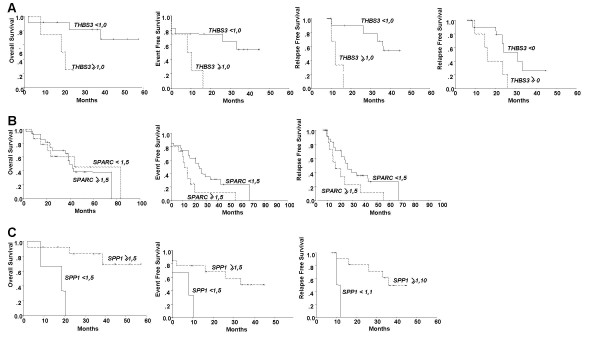
Overall, event-free survival and relapse-free survival analyses of OS patients correlated with gene expression profiling of either biopsy or primary tumor resection onset groups. **A- **Overall, event-free survival and relapse-free survival of biopsy onset group with over-expression of *THBS3*, and relapse-free survival of primary tumor resection onset group with over-expression of *THBS3*. **B- **Overall, event-free survival and relapse-free survival of biopsy onset group with over-expression of *SPARC*.**C- **Overall, event-free survival and relapse-free survival of biopsy onset group with over-expression of the *SPP1 *gene.

A trend for higher expression was observed in samples from patients with metastatic disease at diagnosis (p = 0.058). However, there was no significant correlation with other clinical parameters, such as age at diagnosis, gender, Huvos grade, site of primary tumor, histological type and presence of metastasis at diagnosis. Patients with OS and high levels of expression of *THBS3 *in biopsy samples stratified using the 75^th ^percentile as cutoff value (≥ 1.0), showed worse overall survival (p = 0.03), event-free survival (p = 0.02) and relapse free survival at diagnosis (p = 0.003) (Figure 2A). Patients containing high levels of *THBS3 *expression in surgical specimens stratified using the 50^th ^percentile as cutoff value (≥ 0, over-expression *versus *down-expression), showed worse relapse free survival (p = 0.05) (Figure 2A).

### SPARC mRNA expression in osteosarcoma patients

To confirm *SPARC *over-expression, we performed quantitative real-time PCR on 55 samples from 43 patients. High expression of the *SPARC *gene was found in 51 of 55 tumor samples (96.3%). *SPARC *over-expression did not correlate with the different periods of disease (biopsy, surgery and metastasis) (Figure 1B), presence of metastasis at diagnosis, and the clinico-pathological parameters, such as Huvos grade, age, gender, histological OS subtype, site of primary disease, as well as, the overall survival (p = 0.81) (Figure 2B). Patients with high levels of *SPARC *gene expression stratified using the 75^th ^percentile as cutoff value (≥ 1.5), revealed worse event-free survival (p = 0.03) and relapse free survival (p = 0.07) (Figure 2B).

### SPP1 mRNA expression in osteosarcoma patients

To confirm *SPP1 *over-expression, we performed quantitative real-time PCR on 53 samples from 41 patients. The *SPP1 *gene was over-expressed in 47 of 53 (89%) and under-expressed in 6/53 (11%) of OS samples. In addition, there was a significantly greater level of SPP1 in biopsy samples than in both primary tumor resections and metastatic samples (p = 0.002, p = 0.0002, respectively). However, SPP1 expression levels did not reach significance when primary tumor resection samples were compared to metastases (p = 0.11) (Figure 1C). A recurrent differential level of *THBS3 *expression was also observed when comparing biopsy and primary tumor resection samples from the same patients (n = 7) (p = 0.031). *SPP1 *over-expression did not correlate with the presence of metastasis at diagnosis and the clinico-pathological parameters, such as Huvos grade, age, gender, histological OS subtype and site of primary disease. Patients with OS showing high levels of expression of *SPP1 *in biopsy samples, stratified using the 25^th ^percentile as cutoff value (≥ 1.1), revealed better overall survival (p = 0.001), event-free survival (p = 0.02) and relapse free survival (p = 0.005) (Figure 2C).

## Discussion

In this study, *THBS3*, *SPARC *and *SPP1 *were identified by pathway-focused GEArray^® ^as genes differentially expressed in OS.

The gene for human thrombospondin 3 (*THBS3*) is a member of a family that encodes an extracellular matrix glycoprotein mediating interactions between cells and the extracellular matrix [17,18]. The thrombospondin family of proteins has been implicated in a number of fundamental biological processes: blood coagulation, embryonic development, tissue differentiation, tumor growth and metastases, angiogenesis, nerve development, wound healing, and inflammation [17-19].

Osteonectin (*SPARC*) is a 32kDa protein important for bone calcification [20]; it is a multifunctional glycoprotein that belongs to the matricellular proteins, defined as a group of extracellular regulatory macromolecules relevant to the extracellular matrix structure [21]. It is involved in a variety of biological processes including tissue remodeling, cell adhesion, proliferation, differentiation, matrix synthesis, angiogenesis, tumor cell migration and invasion [22,23].

The *SPP1 *gene (osteopontin) is a non-collagenous bone matrix protein with several functions, such as bone remodeling, angiogenesis, cell adhesion, tumor migration and invasion [24-26]. The functional diversity of osteopontin in bone has many features in common with the repair processes involved in inflammatory responses [27]. It is produced by numerous cell types, osteoblasts, osteoclasts, T lymphocytes, NK cells and epithelial cells from breast, kidney, skin, nerve cells and endothelial cells [24-26].

Invasion and metastases are inherent features of malignant diseases and involve both intercellular and cell-matrix interaction. The molecular mechanisms include transcriptional modulation of adhesive and anti adhesive molecules, proteases and angiogenic factors [28].

As a primary tumor grows, it needs to develop a blood supply that can support its metabolic needs – angiogenesis. These new blood vessels may also provide an escape route by which cells can leave the tumor and enter into the body's circulatory blood system, a process known as "intravasation". The cells need to survive in the circulation until they are arrested in a new organ; where they may extravasate from the circulation into the surrounding tissue. Once in the new site, cells must initiate and maintain growth to form pre-angiogenic micrometastases; this growth must be sustained by the development of new blood vessels in order to form a macroscopic tumor [6].

Thrombospondin 3 is structurally similar to cartilage oligomeric matrix protein (*COMP*/*THBS5*), but its function is unknown. A study in *THBS3*-null mice provided evidence for the role of *THBS3 *in the regulation of skeletal maturation in mice [29]. The role of *THBS1*, the most widely studied gene in this family, in angiogenesis and tumor progression remains controversial. *THBS1 *has been considered an inhibitor of angiogenesis and tumor progression in bladder and lung cancer [30,31]. In contrast, it has also been considered a stimulator of both processes in pancreatic carcinoma, melanoma and hepatocellular carcinoma [32-34]. One study found high *THBS1 *levels in hepatocellular carcinoma and venous invasion in tumors with low levels of vascular endothelial growth factor (VEGF) protein expression [34].

The group with metastasis at diagnosis showed high levels of *THBS3 *expression in biopsy samples, demonstrating the importance of this gene in tumor growth and progression. These data strongly suggest that high *THBS3 *expression can predict worse overall survival, event-free survival and relapse free survival at diagnosis. After chemotherapy, levels of THBS3 declined in some tumors. The groups of patients who maintain over expression have worse relapse free survival, suggesting that keeping *THBS3 *at high levels maintains the capacity of angiogenesis activated by *THBS3*. This is part of the process of tumor progression and perhaps may be used as a prognostic factor and therapeutic target in OS.

To the best of our knowledge, this is the first report about the *THBS3 *gene function as a stimulator of tumor progression in osteosarcoma. Its effects, like those of *THBS1*, may also depend on concentration, type of domain activated or available, and type of receptors present on endothelial cells [33,35,36]. The study of these parameters together with the expression levels of *THBS3 *in OS should be considered, given one's interest in anti-angiogenic agents in the OS treatment protocol.

Osteonectin is produced by osteoblasts and young osteocytes [37]. *SPARC *is expressed at high levels in bone tissue [38].

In this study no differences were observed between the levels of expression in biopsy, primary tumor resection and metastatic site samples, showing that high levels of *SPARC *are not required for tumor progression. However, higher *SPARC *expression (≥ 1.5) has worse event-free survival and shows a tendency to early relapse free survival. This strongly suggests that *SPARC *expression is necessary for tumor growth and maintenance, and may be used as a promising prognostic marker in OS.

Over-expression of *SPARC *promotes tumor growth and was reported in a variety of human malignancies, including melanoma, breast cancer, esophageal cancer and gastric carcinoma [28,39-41], but other reports suggest that decreased *SPARC *expression is associated with increased tumorigenesis and metastases in human ovarian carcinoma and neuroblastoma [42,43]. The promoting or inhibiting effects of osteonectin in different cancers seem dependent upon the cell type, the concentration, and the presence of full-length or proteolytic fragments of osteonectin [44].

Osteopontin (*SPP1*) is a major protein of the extracellular bone matrix, and gene expression is up regulated in specific phases of osteoblastic lineage differentiation [45-47]. *SPP1 *has become of interest in tumorigenesis, and expression of the protein has been observed in human cancer [48].

In this study, we observed that biopsy samples had higher expression of *SPP1 *than samples from primary tumor resection and metastasis, showing that high levels of *SPP1 *are not necessary for tumor progression in OS. At the same time, we noticed that the level of expression is an independent variable from the presence of metastatic disease at diagnosis; these two characteristics indicate that high expression of *SPP1 *is not necessary for tumor progression or metastatic disease in OS. In contrast, high expression of *SPP1 *in other tumors, such as breast cancer, lung cancer, prostate cancer, hepatocellular carcinoma, colon cancer has been associated with tumor progression and metastases [49-52]. Another contrast is that we observed over expression of *SPP1 *correlates with better overall survival, event-free survival and relapse free survival. A study of 57 samples from OS patients by immunohistochemistry reported over expression of *SPP1 *and *VEGF*. However, *SPP1 *levels showed no correlation with overall survival and event-free survival. *SPP1 *is normally expressed in normal bone and, as part of the inflammatory response, participates in the remodeling process of the bone [53]. The functional diversity of *SPP1 *in bone formation and remodeling appears to be related to the fundamental roles of this protein in host defense and tissue repair; in fact, the bone remodeling process has many features in common with the repair processes involved in inflammatory responses [27].

Identification of the molecular determinants of invasion and metastatic pathways will guide the development of a rational strategy for devising specific therapies that target the pathways leading to OS.

## Conclusion

In conclusion, through the screening of arrays we identified genes related to tumorigenesis. Three genes were validated with real-time RT-PCR and correlated with clinico-pathological parameters; whereby the clinical significance of over expression of *THBS3 *for tumor growth and progression, the necessity of *SPARC *for tumor growth and maintenance, and the potential for *SPP1 *as a biological marker of OS were demonstrated.

## Abbreviations

IOP – Instituto de Oncologia Pediátrica

GRAACC – Grupo de Apoio ao Adolescente e à Criança com Câncer

UNIFESP – Universidade Federal de São Paulo

cDNA – complementary deoxyribonucleic acid

PCR – polymerase chain reaction

*THBS3 *– thrombospondin 3

*SPARC *– osteonectin, secreted protein acidic and rich in cysteine

*SPP1 *– osteopontin, secreted phosphoprotein 1

OS – osteosarcoma

*ITGB4 *– integrin beta-4

*CTGF *– connective tissue growth factor

*ITGA5 *– integrin alpha-5

*VIL2 *– villin 2, ezrin, cytovillin

*ITGB2 *– integrin beta-2

*LGALS3 *– galectin-3

*ADAM8 *– a disintegrin and metalloprotease domain 8

*CLU *– clusterin

*FARP1 *– pleckstrin domain-containing protein 1

*CP *– ceruloplasmin

RNA – ribonucleic acid

DEPC water – diethylpyrocarbonate water

*ACTB *– beta-actin

DNA– deoxyribonucleic acid

pUC18 – hypothetical protein PC405478.00.0 [Plasmodium chabaudi chabaudi]

RT-PCR – reverse transcriptase-polymerase chain reaction

Ct – cycle threshold

mRNA – messenger ribonucleic acid

GEO – Gene Expression Omnibus

NK cells – natural killer cells

*COMP*/*THBS5 *– cartilage oligomeric matrix protein

*THBS1 *– thrombospondin 1

*VEGF *– vascular endothelial growth factor

## Competing interests

The author(s) declare that they have no competing interests.

## Authors' contributions

CADT: designed and performed experiments, analyzed data, and wrote manuscript

MY, SCM and CHL: analyzed data and wrote the final manuscript

AMJ: performed statistical analysis as required for the revised manuscript

ASP: selected the cases and worked closely with SRCT

SRCT, JADA, MZ and JAS: designed experiments, analyzed data, and wrote manuscript

JAS: project leader, analyzed data, and wrote manuscript

All authors read and approved the final manuscript.

## Pre-publication history

The pre-publication history for this paper can be accessed here:



## Figures and Tables

**Table 1:** Clinico-pathological description of osteosarcoma patients.

**Parameter**	**Number of patients**	**Parameter**	**Number of patients**
**Age at diagnosis (years)**		**Huvos grade**	
0–10	6	I-II	27
11–20	33	III-IV	7
≥ 21	4	Not informative	9
**Gender**		**Metastasis at diagnosis**	
Male	25	Yes	17
Female	18	No	26
**Histological subtype**		**Site of primary disease**	
Osteoblastic	23	Femur	20
Chondroblastic	7	Tibia	15
Telangiectatic	4	Fibula	2
Giant cells	1	Humerus	4
Other	6	Iliac bone	1
Not informative	2	Other	1

NOTE: Mean follow-up time was 34 months (range from 2 to 83 months)

**Table 2:** Pathway-focused GEArrays^®^: Comparison of three of the up-regulated genes in osteosarcoma samples categorized as metastatic at diagnosis and non-metastatic at diagnosis

**Gene symbol**	**Metastatic at diagnosis**			**Non-metastatic at diagnosis**	
	
	**B/C**	**S/C**	**M/C**	**B/C**	**S/C**
***THBS3***	23.8	53.15	7.1	AE	AE
***SPARC***	13.4	18.6	15.3	6.8	5.0
***SPP1***	7.2	3.7	9.11	4.3	5.3

**B, **sample from biopsy, **S, **sample from primary tumor resection, **M, **sample from metastatic site, **C, **normal bone control, **AE, **absence of expression. The values are expressed as linear ratios against normal bone controls.
